# Genomics and Pharmacogenomics Knowledge, Attitude and Practice of Pharmacists Working in United Arab Emirates: Findings from Focus Group Discussions—A Qualitative Study

**DOI:** 10.3390/jpm10030134

**Published:** 2020-09-18

**Authors:** Azhar T. Rahma, Iffat Elbarazi, Bassam R. Ali, George P. Patrinos, Luai A. Ahmed, Fatma Al Maskari

**Affiliations:** 1 Institute of Public Health, College of Medicine & Health Science, UAE University, Al Ain, P.O. Box 17666, Abu Dhabi, UAE; 201280026@uaeu.ac.ae (A.T.R.); ielbarazi@uaeu.ac.ae (I.E.); luai.ahmed@uaeu.ac.ae (L.A.A.); 2 Department of Pathology, College of Medicine & Health Science, UAE University, Al Ain, P.O. Box 17666, Abu Dhabi, UAE; Bassam.ali@uaeu.ac.ae (B.R.A.); gpatrinos@upatras.gr (G.P.P.); 3 Zayed Canter for Health Sciences, UAE University, Al Ain, P.O. Box 17666, Abu Dhabi, UAE; 4 Department of Pharmacy, School of Health Sciences, University of Patras, 26504 Patras, Greece

**Keywords:** pharmacogenomics, implementation, pharmacists, United Arab Emirates, knowledge, attitude, practice

## Abstract

(1) Background: Genomics and pharmacogenomics are relatively new fields in medicine in the United Arab Emirates (UAE). Understanding the knowledge, attitudes and current practices among pharmacists is an important pillar to establish the roadmap for implementing genomic medicine and pharmacogenomics; (2) Methods: A qualitative method was used, with focus group discussions (FGDs) being conducted among pharmacists working in public and private hospitals in Abu Dhabi Emirate. Snowball sampling was used. Thematic inductive analysis was performed by two researchers independently. NVIVO software was used to establish the themes; (3) Results: Lack of knowledge of genomics and pharmacogenomics among pharmacists was one of the most prominent findings. Therefore, the role of pharmacist in making the right decisions was highlighted to be a barrier for pharmacogenomics implementation in the UAE. Pharmacists have a positive attitude toward pharmacogenomics, but they are preoccupied with concern of confidentiality. In addition, religion and culture shadowed their attitudes toward genetic testing; (4) Conclusions: It is highly recommended to introduce new courses and training workshops for healthcare providers to improve the opportunities for genomics and pharmacogenomics application in the UAE. Pharmacists agreed that the health authorities should take the lead for improving trust and confidence in the system for a better future in the era of genomics and pharmacogenomics.

## 1. Introduction

Pharmacogenomics is defined as the study of the interaction between a drug and any gene, or multiple sites throughout the genome. It is a major approach born out of genomics towards personalized medicine that promotes more precise drug therapy as it tailors the drug and its dose to the genotype of the individual [1]. Pharmacogenomics is currently applied in the treatment of certain cancers, cardiovascular diseases, osteoarticular diseases, gastrointestinal diseases, autoimmune and neuro-psychiatric conditions, and others [2].

Pharmacists’ role in precision and personalized medicine and in pharmacogenomics is highly important. However, despite the agreement on their role in similar manner to clinicians in the decision making for better drugs management, this has not been implemented widely in the UAE. Studies have shown that pharmacists suffer from a lack of information, which impacts on their role and their ability to make the right decisions in pharmacogenetics and pharmacogenomics. Research related to the area of pharmacogenomics has indicated the importance of improving pharmacists’ knowledge and skills for better practice [3,4,5,6,7]. In the United Arab Emirates (UAE), there are no studies that have explored pharmacist’s role, knowledge and attitudes toward genomics and pharmacogenomics. In fact, the sciences of genomics and pharmacogenomics in general are relatively new to the Arab region and in fact are still in their infancy stages [8,9]. A cross-sectional study conducted among pharmacists in Jordan ascertain the theme that the basic knowledge of pharmacogenomics is at a low level though their attitude is optimistic [8]. The congruence stance was identified among pharmacists in Kuwait, in another cross-sectional study among pharmacists and physicians, and only 16.0% stated that they felt assured in implementing pharmacogenetics in their clinics [9]. In the UAE, there is a necessity to explore the road map for the implementation of pharmacogenomics within the routine healthcare systems. Updated medical curricula and pharmacology curricula around the world are nowadays incorporating certain areas that address the science of precision medicine, genomics, genetic testing, and pharmacogenomics [10]. However, it is not clear if this is happening at the moment in the UAE. It is not known if clinicians and pharmacists who are trained either in or outside the UAE are fully aware of this science and competent enough to use it effectively to advance clinical practice and patients’ outcomes. We therefore believe that it is important to investigate pharmacists’ knowledge, attitude and practices to understand the current situation in the UAE. The findings could possibly extrapolate to other healthcare workers in the UAE.

This study aims to explore UAE pharmacists’ knowledge and attitude towards pharmacogenomics to conceptualize a framework for the adoption of pharmacogenomics by pharmacists. The overarching goal of our efforts is to establish the road map for implementing genomic medicine and pharmacogenomics in the country.

## 2. Materials and Methods

A qualitative inductive grounded theory approach informed by the Corbin and Strauss (2008) methodological pathway was followed to develop a theory related to the field of pharmacogenomics in the UAE [11]. Four focus group discussions were conducted to explore the knowledge, attitude and perception of registered pharmacists working in the UAE toward genomics and pharmacogenomics. Pharmacists were invited through hospitals, clinics and community pharmacies and using snowball techniques. Inclusion criteria included any registered pharmacists of any nationality working either in private or public settings and in any health care setting (tertiary hospitals, health clinics or community pharmacies) as either outpatient, inpatient or clinical pharmacists. Participants were invited in person, by telephone and via email. All who agreed to participate received an official invitation via email including details of the meetings as well as the information sheet of the study and time and location of the meeting. A reminder email and messages were sent one week before the session and repeated 24 h and 2 h before the session. The sessions were conducted over weekends at the College of Medicine and Health Sciences of the United Arab Emirates University to ensure that participants attend the meeting.

The four focus group discussions were conducted and moderated by IE and AR. Each session lasted 90 min. Saturation was reached after the fourth focus group discussion.

Researchers followed an interview guide with questions and prompts that had been revised by experts in the field of qualitative studies, public health as well as genomic medicine. All four focus group sessions were audio taped during the sessions and field notes were recorded during and after the focus group sessions. This study had been approved by social science research ethics committee of United Arab Emirates University (UAEU) ERS_2017_5671. Participants were asked to read the information sheet of the study as well as to sign the consent form before starting the discussion.

A verbatim transcription for all focus group sessions was reviewed by two researchers and then was returned for random participants for comments and/or corrections to ensure credibility and reflexivity. All four focus group sessions were coded and themes were extracted. Inter-coder reliability was assured. The transcription was uploaded on Nvivo 12 (Windows version) for analysis to extract themes and visualize the findings.

## 3. Results

The reporting of the findings follows the consolidated criteria for reporting qualitative research (COREQ) checklists [12] as well as Lincoln and Guba’s evaluation criteria [13].

Participants’ demographics are presented in Supplementary Table S1. More than half of the participants were expatriates, females and above 30 years old. The sample included pharmacists working in the inpatient setting and the outpatient setting as well as clinical pharmacists and pharmacy residents. Some participants have postgraduate qualifications, and some have experience working outside the UAE, with most of them having more than 11 years of experience. The vast majority of the participants stated that they did not receive formal education about genomics and pharmacogenomics at a higher education level.

Themes extracted were based on our interview guide, which explored knowledge, attitude and current practice, future direction and needs in the area of genomics and pharmacogenomics. However, many themes have emerged from the focus group sessions that we have classified as emerging themes. Below we will present the main themes in three different sections (knowledge, attitude, practice and future directions), then we will present the emerging themes: Pharmacists’ role and power, skills, trust and blame as well as cultural and religious beliefs.

### 3.1. Main Themes

#### 3.1.1. Knowledge

The knowledge of pharmacists about genomic medicine and pharmacogenomics in particular had been identified as a main theme. Moreover, sub-themes extracted included knowledge about genomics and pharmacogenomics, practice, services, as well as sources of information and coverage of the costs of testing (Supplementary Figure S1)

##### Knowledge about the Science of Genomics and Pharmacogenomics, Practice, and Services

During the focus group discussion, participants were asked to rank their knowledge about pharmacogenomics on a scale from 0 to 5 (0 is poor knowledge and 5 is excellent knowledge) (Supplementary Figure S2). More than third of the pharmacists rated their knowledge of genomics and pharmacogenomics as poor; one pharmacist said:

“*Actually, we didn’t hear about genomics and pharmacogenomics before this invitation*”

FG2M9.

Most of the participants indicated that they are not sure where genetic testing is conducted in the UAE. Moreover, most of them had no knowledge about where to locate tests results in patients’ electronic records. In addition, they felt there is poor dissemination of information from stakeholders to consumers and healthcare providers.

“*They [stakeholders] are not sharing it with staff so we do not know*”

FG4M12.

When we asked them if they are aware of the application of genomic medicine or pharmacogenomics in their hospitals, they were unsure; one pharmacist pointed out that his hospital is conducting a test for Glucose-6-Phosphate Dehydrogenase (G6PD) deficiency but was not sure if this is a pharmacogenetic test.

##### Knowledge about Sources of Information Related to Genomics and Pharmacogenomics

Most of the senior pharmacists in our sample did not receive formal education related to genomics and pharmacogenomics at universities and the few (nine pharmacists) who studied pharmacogenomics before classified their knowledge as being poor.

“*we took a course on pharmacogenomics but did not benefit even professor was lost*”

FG1F4.

Even some fresh graduates from a semi-governmental university in the UAE stated that they did not receive formal education about genomics or pharmacogenomics at all during their pharmacy bachelors’ years.

As for the most frequently utilized sources of information regarding genomics and pharmacogenomics among the participants, Google search and YouTube videos were the most utilized sources. However, it was noted that all clinical pharmacists in the focus group discussion indicated that databases and trusted organizations (outside the UAE) are their sources of information, believing that there is a gap in the available resources by the UAE health authorities. A couple of participants indicated that the sources of their information are enough to give them the needed knowledge.

“*I watched a video on YouTube last night about pharmacogenomics, so I know what I am talking about…*”

FG2M9.

Pharmacists agreed that they lack competency in interpreting genomics and pharmacogenomics test reports, and they were not aware that the drug leaflets contain sections related to pharmacogenomics.

“*if you didn’t invite me to this focus group I wouldn’t know this piece of information or any other knowledge discussed. So I think there is no awareness*”

FG1M4.

##### Knowledge about Cost and Coverage of Genetic Tests

Outpatient pharmacists in the focus group discussion were more aware about the cost and coverage of genetic testing. An example used by participants is the case of patients with cystic fibrosis and how the insurance companies are mandating and covering the genetic tests before the initiation of the therapy. Another example is anticoagulant coverage by health insurance.

“*We know many cases where patient get stents and the inpatient cost is covered by insurance, but when discharged and they have to pay they refuse to take their anticoagulant medications (Plavix) and they are readmitted again with thrombosis*”

FG4M13.

#### 3.1.2. Attitudes

The second major theme underscored is the attitude of pharmacists toward genomics and pharmacogenomics. We identified the following sub-themes: benefits of pharmacogenomics, disclosure of genetic testing and biobanking, and the implementation of pharmacogenomics within services (Supplementary Figure S3).

##### Pharmacists’ Attitude about Benefits of Genomics and Pharmacogenomics

Most participants showed an overall positive attitude toward pharmacogenomics, despite their lack of knowledge in the sciences of pharmacogenomics and genomics. Nevertheless, some of them consider pharmacogenomics as a science fiction and an area that lacks solid evidence.

“*A lot of things was pending investigations, everything was not clear, as I just said earlier it is uncharted territory, so a lot of new thing was… introduced and nothing basic, so I think it is a good branch and I am enthusiastic about it but still it is a new branch, so no one had solid things to give it to you, it was like watching something that will happen in the future*”

FG2F8.

However, some had negative attitude toward the importance of learning and the use of pharmacogenomics:

“*What is the point of learning something that will be implemented 20 years later*”

FG1M5.

“*Why to waste money in something that will not benefit me*”

FG4M14.

“*What is the point of knowing about it if we are not going to practice it. In UAE, there is no market for genetics*”

FG1M2.

##### Pharmacists’ Attitude toward Disclosure of Genetic Test Results and Biobanking

Pharmacists had a mixed attitude toward knowing the results of their whole genome sequencing; some wanted to know so they can lead a healthy lifestyle and keep an eye on research, looking for interventions. The others did not want to know out of fear and religious belief as well as its impact on their social and family life.

“*Leave it to God*”

FG2M9.

“*take the example of Angelina Jolie she was doing so fine before the test then after that she lost weight and get divorced, so doing the test was bad for her!*”

FG3F13.

However, when it was related to their children, there was a consensus on the desire to know how to protect their children.

“*From my personal experience with vision problem and my mother have cancer disease I want to protect my children from the disease I have or my mother have it. I see how my mother suffer with cancer and whatever it takes not to go through that suffering will drive you to protect yourself or your children.*”

FG1M2.

When we explored their attitude toward participating in biobanking, there were mixed attitudes, with some being supportive of the idea as they believed it is vital to research and consolidate community health, while others did not show any support for it. For example, one expatriate pointed out that biobanking should be directed only toward UAE nationals, rather than expatriates since the expatriates may leave at any time, affecting the follow up and research logistics.

##### Pharmacists’ Attitude toward the Implementation Mechanism of Genomic Medicine and Pharmacogenomics

Pharmacists did not agree on the proper and ideal mechanism of implementing genomic medicine and pharmacogenomics in the UAE. Some pharmacists advocated a preemptive pharmacogenetic testing approach, which seeks proactive testing and obtaining the results of the genetic test at the time of prescribing, and their arguments were:

“*Test will be cost effective, because you will do it once, for example most of the drugs are metabolized by the Cytochrome P450 (CYP450) enzyme. So by doing this test alone we will be able to identify poor or rapid metabolizers which will protect them from the harm of certain medications. Lets say the test will cost 200 $ once per life and it will stay in the chart of the patients for long time, and this is for value for expensive medications.*”

FG4M13.

On the other hand, other participants were advocates of the reactive pharmacogenetic testing approach, in which specific drug–gene tests will be requested at the time of dispensing:

“*when the guidelines for hypertension was to use diuretics first, beta blockers second etc the old guidelines, they said for Africans you should go for calcium channel blockers. that was good, but do I need to do genome test that will cost me thousand dollars to know ? I don’t think so. simply you can use the diuretics for couple of days if it is not working then I will put beta blocker, if I have enough numbers of patient that will prove the theory that this medication is not effective in this ethnicity at that point I will go for genetic test, but I will not go before that.*”

FG4M12.

### 3.2. Emerging Themes

#### 3.2.1. Power

One emerging theme identified by this study was the lack of power and feeling of powerlessness of pharmacists in making decisions related to pharmacogenomics.

“*Even the stakeholders will not focus on pharmacists, their main focus is physicians. Pharmacists are always out of the picture in any decision*”

FG1M2.

Moreover, they linked that attitude to stakeholders’ influence, no clear guidelines about genomics and pharmacogenetics and their roles in the implementation.

“*we can’t do it on our own, we cannot make decisions*”

FG3F14.

Only clinical pharmacists working in oncology services could envision their role, but they disclosed that the knowledge gap hinders this role and that they do not have the power.

#### 3.2.2. Trust and Responsibility

Another emerging theme is the fear of losing their patients’ trust. Participants stated that since they do not have the knowledge of genomics and pharmacogenomics, they worry that they may lose the trust and rapport that they have with their patients.

“*To be honest for us currently as a healthcare provider who don’t know much about genomics and pharmacogenomics, so how we will initiate the talk with the patient*”

FG3M11.

They also exhibited concerns about trusting the system in terms of confidentiality and they worried that they may lose their jobs based on their genetic test results.

“*I will never do the genetic test, if they find out that I have certain disease they may fire me from my job, I will never do it even if they said there is confidentiality, there is no law and I will not take the risk*”

FG4M12.

Pharmacists declared that they do their own patient counselling because they have the skills, as well as being trained in their workplace about how to conduct counselling. Nevertheless, they questioned their competency to do counselling about pharmacogenomics to the patients when they do not have the knowledge of pharmacogenomics. They worried that they may lose the trust and rapport that they have with their patients.

#### 3.2.3. Fatalism and Stigma

Pharmacists believed that it is all is in God’s hand and nothing they can do will change destiny. Some have revealed doubts that religion might constitute a barrier for the implementation of genomic medicine in the UAE.

“*who are we to interfere in destiny*”

FG2F8.

Pharmacists assumed that culture had a powerful impact on the adoption of genomic medicine more than religion.

“*culture is one of the biggest challenges and barriers and should be factored in while drafting laws and policies*”

FG2M6.

Nevertheless, they perceived culture as dynamic and they supported that by comparing the era of genomic medicine to the era of organ transplant and in vitro fertilization and how the community were opposers and now they are adopters.

The fear of stigma was not exclusive to the UAE; even pharmacists from other nationalities fear the labeling and stigmatizing of their families with certain genetic diseases. One pharmacist stated:

“*……they change the law, so the couples will not do the premarital test at the same time, we will start with the man and based on the results of his test they will decide if to do the test for the lady……………see they change the law for the effect of the culture and the fear of stigma*”

FG3F22.

## 4. Discussion

Lessons from the implementation of genomic medicine and pharmacogenomics worldwide suggests that gauging the knowledge and attitude of healthcare providers is a prerequisite to exploring the road map for the implementation of pharmacogenomics and possibly genomic medicine within the routine healthcare systems. Nevertheless, it is not clear if this is happening now in the UAE. The novelty of our findings is that it is the first qualitative study in the UAE that will allow stakeholders to follow a clear pathway/framework for the adoption of genomics and pharmacogenomics in clinical practice. Our findings provide multilayers of factors and inputs like knowledge, attitude, perception, ecological factors, and power that will be useful in implementing pharmacogenomics in the UAE. The pharmacists indicated that the power is with the leaders of the healthcare system, and we therefore suggest a further study into the stakeholders’ knowledge and attitudes towards implementing pharmacogenomics and genomic medicine in the country.

Several studies evaluated the knowledge and attitude of pharmacists toward genomics and pharmacogenomics world-wide and our study is the first to do so in the UAE. Despite the geographical spaces, pharmacists shared similar attitudes and concerns toward pharmacogenomics [8,9,14,15,16,17,18,19,20,21,22,23]. In our sample, the knowledge of pharmacists who worked or studied outside the UAE did not differ from those who worked or studied in the UAE. In addition, being a fresh graduate did not influence the level of knowledge of pharmacists about genomics and pharmacogenomics and that is in contrast with what Pisanu et al., reported; that new graduates had better knowledge in pharmacogenomics in comparison to senior graduates [19]. We observed pseudo-knowledge as pharmacists in our sample were mixing between genomic medicine and genetic engineering or screening and that can be attributed to the poor knowledge and the gap in the curriculum. This calls for incorporating genomics and pharmacogenomics education more effectively in the current training programs.

Yau A. et al., assessed the practice of genomic medicine and pharmacogenomics by pharmacists as well as their knowledge and attitude in a systematic review and they concluded that pharmacists ought to be taught how to read genetic test reports and act upon them [24]. Our findings are in accordance with that conclusion, as despite the positive attitude that pharmacists in our sample had toward genomic medicine and pharmacogenomics, they ranked their knowledge level as poor or fair.

The American Society of Health-System Pharmacists (ASHP) highlighted the responsibilities, roles and functions of the pharmacist in the pharmacogenomics era [25]. However, limited studies assessed pharmacists’ health literacy skills and factors prominent to the adoption of pharmacogenomics. A study by Romangnoli et al., used a qualitative method to assess the resource requisite of the pharmacists in Pittsburgh, United States, and they concluded that whenever a pharmacogenomics tool will be designed, pharmacist’s requirements is an essential step to be factored in, particularly in terms of translation of the genetic test. [26] We identified a gap in the tools that pharmacists use to seek information. Most pharmacists in this study identified internet surfing, Google and YouTube as their main source of information, except for a few clinical pharmacists who navigate databases and scientific journals and stated that the internet may have unscientific information. It is worth mentioning that these skills are dynamic in nature and are an integral component of the framework of the pharmacogenomics literacy of pharmacists. A way to close this gap can happen if authorities and policy makers provide official clinical practice pathways and references for healthcare providers in the UAE.

Pharmacists in this study have agreed that the decision to implement genomics and pharmacogenomics in the UAE is in the hands of stakeholders. A wide range of papers discussed the role of stakeholders and the gaps that hinder the adoption of genomic medicines [14,27,28].

Fatalism is one of the emergent themes that we coded, Elbarazi et al. had investigated the influence of religion on opinions related to health in the UAE and they highlighted the necessity of having a personalized set of religious values in decision making [29]. Nevertheless, our study is the first to shed light on the implication of religion on the adoption of genomic medicine among healthcare providers. Pharmacists in our sample were advocates of genetic testing to their offspring and they attributed that to their maternal and paternal instincts of protecting their children; these findings are parallel to the findings of Hallowell et al. in which participants value the genetic tests in promoting the health of their relatives, particularly their children [30].

Pharmacists did not agree on the proper and ideal mechanism of implementing genomic medicine and pharmacogenomics in the UAE. Some pharmacists advocated a preemptive pharmacogenetic testing approach, which seeks proactive testing and obtaining the results of the genetic test at the time of prescribing [31]. On the other hand, other participants were advocates of the reactive pharmacogenetic testing approach, in which specific drug–gene tests will be requested at time of dispensing [32].

Pharmacists perceived that a multidisciplinary team of physician, pharmacist and genetic counsellor may be the best approach to tackle pharmacogenomic communication in the light of the current scene of the lack of knowledge, workload and shortage of personal [33].

Myriad studies postulated the feasibility of pharmacists’ role in implementing pharmacogenomics at bed side and health settings. A pilot study by Bank PC et al. in the Netherlands underscored the efficient role of community pharmacists in recommending intervention based on the drug–gene of the patients, and these recommendations were acknowledged by the clinicians in 88.7% of the patients [34].

Stark Z. et al. advocated the global liability of transforming genomics into healthcare. In their paper, they delignated the different implementation strategies taken by 15 countries, namely: the UK, France, Australia, Saudi Arabia, Turkey, the US, Estonia, Denmark, Japan, Qatar, Switzerland, the Netherlands, Brazil, Finland and China [35]. These strategies and initiatives can be tools for the adoption of genomic medicine and pharmacogenomics in the UAE to avoid reinventing the wheel and squandering resources.

Pharmacists in the UAE are thirsty for resources and tools to foster their competency in genomics and pharmacogenomics. The Implementing GeNomics In praTticE (IGNITE Toolbox) is one of many open peer reviewed resources that consolidate the knowledge and implantation efforts of pharmacists and other healthcare providers. The Clinical Pharmacogenetics Implementation Consortium (CPIC) provides guidelines on converting genetics results to actionable interventions [36]. This is accompanied by the PharmGKB, which grants knowledge incorporated in pathways [37].

Scholars are equipping healthcare providers with tools to overcome the gap in their knowledge. Zarei S. et al. coined a web-based pharmacogenomics search instrument for the pharmacogenomics of drugs used in anesthesia [38]. The Genotype-Tissue Expression (GTEx) Consortium is another resource [39].

Ziegelstein RC, in his commentary, diagnosed personomics as the gap of the adoption and evolution of personalized medicine. In consonance with this punchline, we hypothesize that the healthcare providers and, more specifically, pharmacists are rooted in the personomics concept. Moreover, addressing their knowledge, attitude and perception will reshape the face of medicine in the country [40].

As recommended by the 9th Santorini Conference conducted in Greece, establishing a research link between academics and businesses will bridge the gaps and chasm in the roadmap for full implementation of genomic medicine and pharmacogenomics [41]. These recommendations can guide the UAE in its strategy for implementing genomic medicine and pharmacogenomics.

The strength of our study is that to our knowledge, this is the first qualitative study to be conducted among pharmacists in the UAE that discusses the adoption of genomics and pharmacogenomics in the UAE. The qualitative nature of the study allows us to dig deeper and enables us to view a comprehensive picture. The limitations of our study were that we could not have higher representation of the community pharmacists; they declined the participation in our focus group due to workload shifts and difficulties in obtaining manager approval. We attempted to conduct one focus group discussion near their workplace and not on a weekend but that did not help. Another limitation is the lack of representation of all the seven cities of the UAE; despite the snowballing sampling technique, we could not have enough representation from cities other than Abu-Dhabi city. We found difficulty in recruiting pharmacists to participate in the focus group discussions; forty-three invitations were rejected, mainly due to lack of knowledge about the topic.

## 5. Conclusions

We believe that our findings can be set as the starting point for establishing the roadmap for the implementation of genomic medicine and pharmacogenomics among pharmacists, as they address a plethora of factors related to pharmacists, including individual factors and ecological factors. It is highly recommended to introduce new courses and training workshops or even accredited certifications for pharmacists to improve the chances of genomics and pharmacogenomics application in the UAE, as knowledge is an essential and dynamic pillar for the implementation of genomic medicine and pharmacogenomics. Pharmacists agreed that the government need to work on improving trust and confidence in the system for a better future in the era of genomics, genetic testing, and pharmacogenomics. For personalized medicine to take center stage in the UAE, we recommend tackling its implementation using a multidisciplinary approach. This should start with the knowledge of pharmacists and all healthcare providers who are the steering wheel of the adoption of this evolving science. That can be coupled with building the infrastructure in the healthcare systems and creating robust guidelines and research databases. Beside the aforementioned recommendations, we propose addressing the ecological factors that may hinder the elite implementation of genomic medicine and pharmacogenomics in the UAE.

We recommend: 1. Inaugurating mandatory pharmacogenomics’ competency for pharmacists in the UAE. 2. Lunching courses and workshops in collaboration with universities. 3. Encompassing pharmacists as a key stakeholder in the piloting phase of the implementation of pharmacogenomics. 4. Constructing algorithms and guidelines to guide pharmacists. 5. Fostering an electronic decision support system and configurating it with the pharmacists’ interface. 6. Assembling regular focus group discussions with pharmacists to identify and address their needs and challenges.

## Supplementary Materials

The following are available online at https://www.mdpi.com/2075-4426/10/3/134/s1, Table S1: Participant demographics, Figure S1: Main themes and subthemes of the knowledge of the pharmacists in United Arab Emirates toward genomic medicine and pharmacogenomics, Figure S2: The rating of the perceived knowledge of the pharmacists in the United Arab Emirates toward pharmacogenomics, Figure S3: Main themes and subthemes of the attitude of the pharmacists in United Arab Emirates toward genomic medicine and pharmacogenomics.

## References

[1] Lauschke V.M., Ingelman-Sundberg M. (2020). Emerging strategies to bridge the gap between pharmacogenomic research and its clinical implementation. NPJ Genom. Med..

[2] Mini E., Nobili S. (2009). Pharmacogenetics: Implementing personalized medicine. Clin. Cases Min. Bone Metab..

[3] Kennedy M.J. (2018). Personalized medicines–are pharmacists ready for the challenge?. Integr. Pharm. Res. Pract..

[4] Owen J.A. (2011). Integrating pharmacogenomics into pharmacy practice via medication therapy management: American Pharmacists Association. J. Am. Pharm. Assoc..

[5] McCullough K.B., Formea C.M., Berg K.D., Burzynski J.A., Cunningham J.L., Ou N.N., Rudis M.I., Stollings J.L., Nicholson W.T. (2011). Assessment of the pharmacogenomics educational needs of pharmacists. Am. J. Pharm. Educ..

[6] Roederer M.W., Kuo G.M., Kisor D.F., Frye R.F., Hoffman J.M., Jenkins J., Weitzel K.W. (2017). Pharmacogenomics competencies in pharmacy practice: A blueprint for change. J. Am. Pharm. Assoc..

[7] Benzeroual K.E., Shah B., Shinde S. (2012). Pharmacogenomics: Assessing educational exposure, confidence in knowledge and training elements of pharmacists. Pers. Med..

[8] AlEjielat R., Ejielat Z., Andrawes S., Mhaidat N.M. (2016). An evaluation of the knowledge, opinions, expectations and concerns toward pharmacogenomics among Jordanian pharmacists. Pers. Med..

[9] Albassam A., Alshammari S., Ouda G., Koshy S., Awad A. (2018). Knowledge, perceptions and confidence of physicians and pharmacists towards pharmacogenetics practice in Kuwait. PLoS ONE.

[10] Murphy J.E., Green J.S., Adams L.A., Squire R.B., Kuo G.M., McKay A. (2010). Pharmacogenomics in the curricula of colleges and schools of pharmacy in the United States. Am. J. Pharm. Educ..

[11] Corbin J., Strauss A. (2008). Strategies for qualitative data analysis. Basics of Qualitative Research: Techniques and Procedures for Developing Grounded Theory.

[12] Tong A., Sainsbury P., Craig J. (2007). Consolidated criteria for reporting qualitative research (COREQ): A 32-item checklist for interviews and focus groups. Int. J. Qual. Health Care.

[13] Lincoln Y.S., Guba E.G. (1986). But is it rigorous? Trustworthiness and authenticity in naturalistic evaluation. New Dir. Program Eval..

[14] Tai C.G., Harris-Wai J., Schaefer C., Liljestrand P., Somkin C.P. (2018). Multiple Stakeholder Views on Data Sharing in a Biobank in an Integrated Healthcare Delivery System: Implications for Biobank Governance. Public Health Genom..

[15] Abdela O.A., Bhagavathula A.S., Gebreyohannes E.A., Tegegn H.G. (2017). Ethiopian health care professionals’ knowledge, attitude, and interests toward pharmacogenomics. Pharm. Pers. Med..

[16] Muzoriana N., Gavi S., Nembaware V., Dhoro M., Matimba A. (2017). Knowledge, Attitude, and Perceptions of Pharmacists and Pharmacy Students towards Pharmacogenomics in Zimbabwe. Pharmacy.

[17] Tuteja S., Haynes K., Zayac C., Sprague J.E., Bernhardt B., Pyeritz R. (2013). Community pharmacists ‘attitudes towards clinical utility and ethical implications of pharmacogenetic testing. Pers. Med..

[18] De Denus S., Letarte N., Hurlimann T., Lambert J.-P., Lavoie A., Robb L., Sheehan N.L., Turgeon J., Vadnais B. (2013). An evaluation of pharmacists’ expectations towards pharmacogenomics. Pharmacogenomics.

[19] Pisanu C., Tsermpini E.-E., Mavroidi E., Katsila T., Patrinos G.P., Squassina A. (2014). Assessment of the pharmacogenomics educational environment in Southeast Europe. Public Health Genom..

[20] Karuna N., Tragulpiankit P., Mahasirimongkol S., Chumnumwat S. (2020). Knowledge, attitude, and practice towards pharmacogenomics among hospital pharmacists in Thailand. Pharm. Genom..

[21] Weinstein S., Carroll J.C., Jukic S., McGivney M.S., Klatt P. (2020). Perspectives of a pharmacist-run pharmacogenomic service for depression in interdisciplinary family medicine practices. J. Am. Coll. Clin. Pharm..

[22] Berenbrok L.A., Hart K.M., McGrath S.H., Coley K.C., McGivney M.A.S., Empey P.E. (2019). Community pharmacists’ educational needs for implementing clinical pharmacogenomic services. J. Am. Pharm. Assoc..

[23] Nagy M., Lynch M., Kamal S., Mohamed S., Hadad A., Abouelnaga S., Aquilante C.L. (2020). Assessment of healthcare professionals’ knowledge, attitudes, and perceived challenges of clinical pharmacogenetic testing in Egypt. Pers. Med..

[24] Yau A., Abd Aziz A.B., Haque M. (2015). Knowledge, Attitude and Practice Concerning Pharmacogenomics among Pharmacists: A Systematic Review. J. Young Pharm..

[25] American Society of Health-System Pharmacists (2015). ASHP statement on the pharmacist’s role in clinical pharmacogenomics. Am. J. Health-Syst. Pharm..

[26] Romagnoli K.M., Boyce R.D., Empey P.E., Adams S., Hochheiser H. (2016). Bringing clinical pharmacogenomics information to pharmacists: A qualitative study of information needs and resource requirements. Int. J. Med. Inform..

[27] Bush W.S., Bailey J.N.C., Beno M.F., Crawford D.C. (2019). Bridging the Gaps in Personalized Medicine Value Assessment: A Review of the Need for Outcome Metrics across Stakeholders and Scientific Disciplines. Public Health Genom..

[28] Snyder S.R., Mitropoulou C., Patrinos G.P., Williams M.S. (2014). Economic evaluation of pharmacogenomics: A value-based approach to pragmatic decision making in the face of complexity. Public Health Genom..

[29] Elbarazi I., Devlin N.J., Katsaiti M.-S., Papadimitropoulos E.A., Shah K.K., Blair I. (2017). The effect of religion on the perception of health states among adults in the United Arab Emirates: A qualitative study. BMJ Open.

[30] Hallowell N., Alsop K., Gleeson M., Crook A., Plunkett L., Bowtell D., Mitchell G., Young M.-A., Australian Ovarian Cancer Study G. (2013). The responses of research participants and their next of kin to receiving feedback of genetic test results following participation in the Australian Ovarian Cancer Study. Genet. Med..

[31] Keeling N.J., Rosenthal M.M., West-Strum D., Patel A.S., Haidar C.E., Hoffman J.M. (2019). Preemptive pharmacogenetic testing: Exploring the knowledge and perspectives of US payers. Genet. Med..

[32] Arwood M.J., Chumnumwat S., Cavallari L.H., Nutescu E.A., Duarte J.D. (2016). Implementing pharmacogenomics at your institution: Establishment and overcoming implementation challenges. Clin. Transl. Sci..

[33] Wurcel V., Cicchetti A., Garrison L., Kip M.M.A., Koffijberg H., Kolbe A., Leeflang M.M.G., Merlin T., Mestre-Ferrandiz J., Oortwijn W. (2019). The value of diagnostic information in personalised healthcare: A comprehensive concept to facilitate bringing this technology into healthcare systems. Public Health Genom..

[34] Bank P.C.D., Swen J.J., Schaap R.D., Klootwijk D.B., Baak–Pablo R., Guchelaar H.-J. (2019). A pilot study of the implementation of pharmacogenomic pharmacist initiated pre-emptive testing in primary care. Eur. J. Hum. Genet..

[35] Stark Z., Dolman L., Manolio T.A., Ozenberger B., Hill S.L., Caulfied M.J., Levy Y., Glazer D., Wilson J., Lawler M. (2019). Integrating genomics into healthcare: A global responsibility. Am. J. Hum. Genet..

[36] Cavallari L.H., Beitelshees A.L., Blake K.V., Dressler L.G., Duarte J.D., Elsey A., Eichmeyer J.N., Empey P.E., Franciosi J.P., Hicks J.K. (2017). The IGNITE Pharmacogenetics Working Group: An opportunity for building evidence with pharmacogenetic implementation in a real-world setting. Clin. Transl. Sci..

[37] Thorn C.F., Klein T.E., Altman R.B. (2013). PharmGKB: The pharmacogenomics knowledge base. Pharmacogenomics.

[38] Zarei S., Costas Y., Orozco G., Zaydlin M., Mirtar A., Abouali M., Diaz-Marty C., Akhlaghipour G., Altamirano P.F., Cardona A.R.G. (2020). A Web-Based Pharmacogenomics Search Tool for Precision Medicine in Perioperative Care. J. Pers. Med..

[39] Keen J.C., Moore H.M. (2015). The Genotype-Tissue Expression (GTEx) Project: Linking clinical data with molecular analysis to advance personalized medicine. J. Pers. Med..

[40] Ziegelstein R.C. (2017). Personomics: The missing link in the evolution from precision medicine to personalized medicine. J. Pers. Med..

[41] Visvikis-Siest S., Gorenjak V., Stathopoulou M.G., Petrelis A.M., Weryha G., Masson C., Hiegel B., Kumar S., Barouki R., Boerwinkle E. (2018). The 9th Santorini Conference: Systems Medicine, Personalised Health and Therapy.“The Odyssey from Hope to Practice”, Santorini, Greece, 30 September–3 October 2018. J. Pers. Med..

