# Supplementary material for: Genomics and Pharmacogenomics Knowledge, Attitude and Practice of Pharmacists Working in United Arab Emirates: Findings from Focus Group Discussions—A Qualitative Study
Source: J Pers Med. 2020 Sep 18;10(3):134. doi: 10.3390/jpm10030134 (PMC7563679; doi:10.3390/jpm10030134)
Supplement: Supplementary file 1 [file jpm-10-00134-s001.pdf]

Table S1: Participant demographics.

	**Number of pharmacists participated= 38**
**Age :**	
<30 years old	10
>31 years	28
**Gender:**	
Male	14
Female	24
**Total years of experiences:**	
<4 years	6
5- 10 years	4
> 11years	28
**Studied Pharmacogenomics in college:**	
Yes	9
No	29
**Current position:**	
Pharmacist (outpatient)	7
Pharmacist (inpatient)	15
Clinical Pharmacist	6
Pharmacy supervisor	5
Resident	4
Community pharmacist	1
**Type of facility:**	
Tertiary care Hospital	34
Secondary care Hospital	0
Health clinic	3
Other	1
**Facility operated by:**	
Government.	35
Non-Government	3
**Nationality:**	
Locals	7
Non-locals	31
**Qualification:***	
BSc	18
Master	9
Pharm.D	8
Board Certified	12
**Practiced outside United Arab Emirates:**	
Yes	23
No	15
Number of declined participation	43

*Sum exceeds 100% as participants can pick more than one choice.

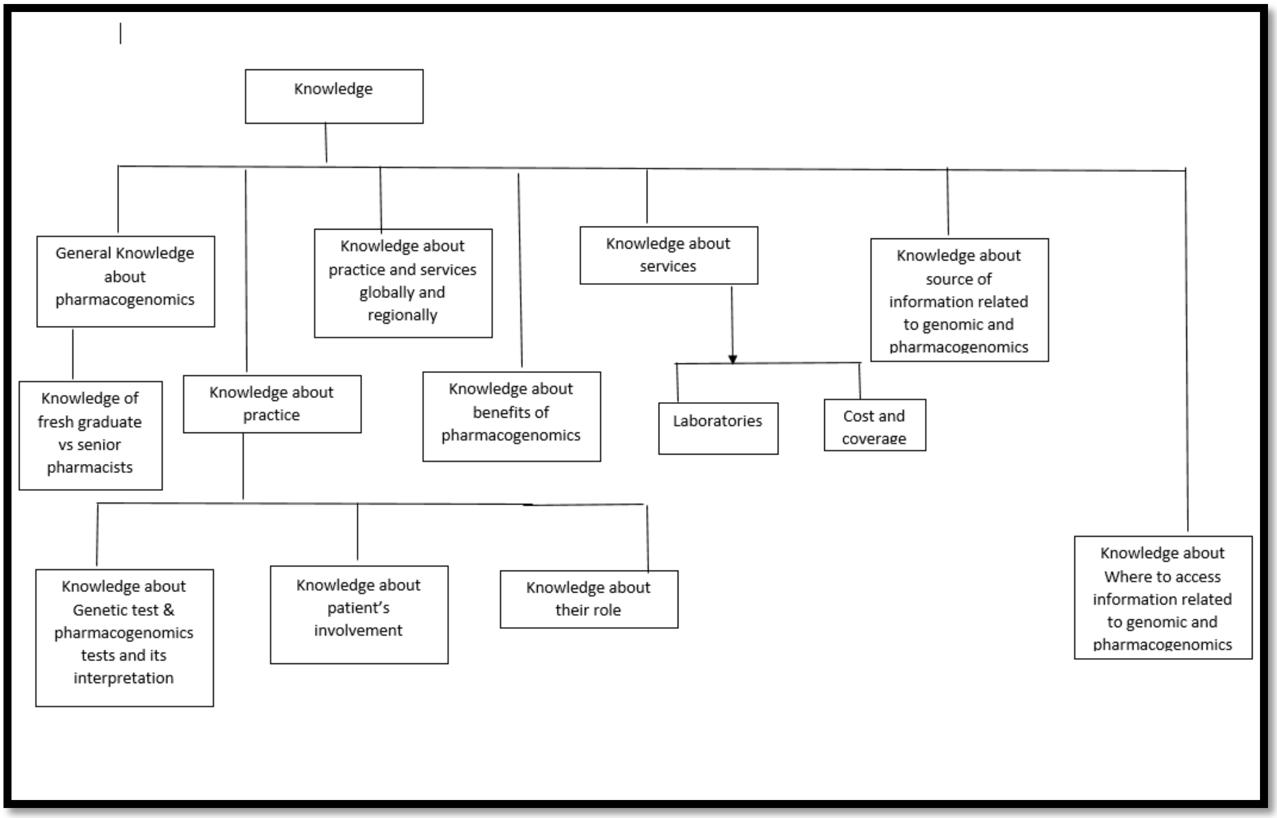

Figure S1. Main themes and subthemes of the knowledge of the pharmacists in United Arab Emirates toward genomic medicine and pharmacogenomics

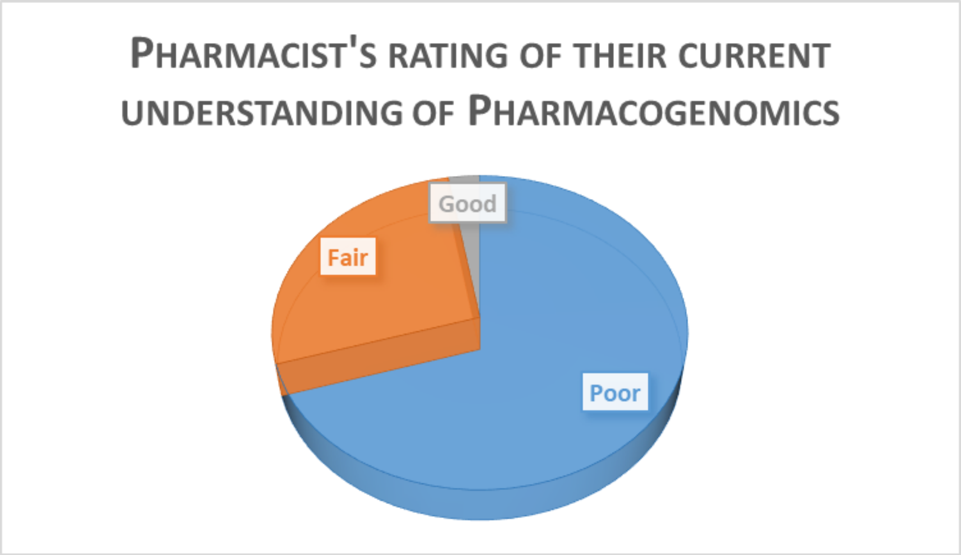

Figure S2. The rating of the perceived knowledge of the pharmacists in United Arab Emirates toward pharmacogenomics

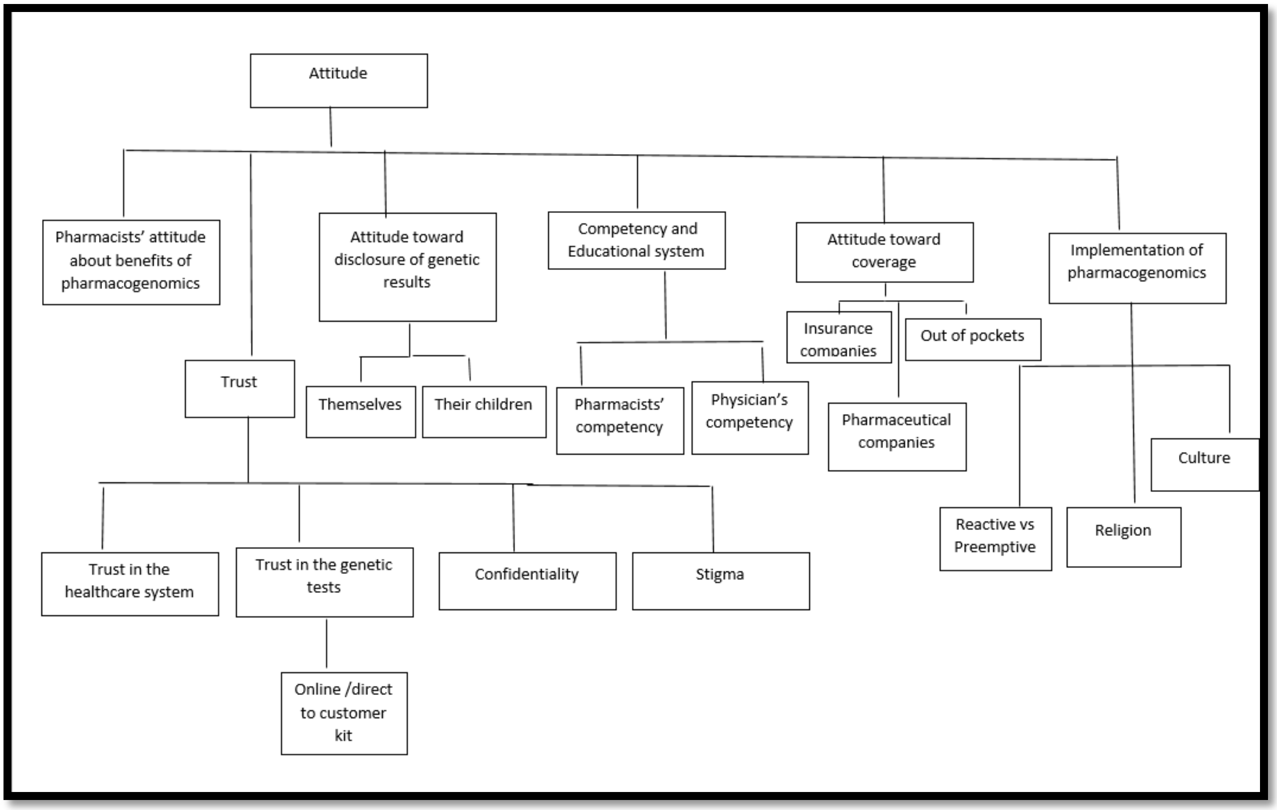

Figure S3. Main themes and subthemes of the attitude of the pharmacists in United Arab Emirates toward genomic medicine and pharmacogenomics
